# Assessing the uptake of incentivised physical health checks for people with serious mental illness: a cohort study in primary care

**DOI:** 10.3399/BJGP.2023.0532

**Published:** 2024-06-25

**Authors:** Maria Ana Matias, Rowena Jacobs, María José Aragón, Luis Fernandes, Nils Gutacker, Najma Siddiqi, Panagiotis Kasteridis

**Affiliations:** Centre for Health Economics, University of York, York, UK.; Centre for Health Economics, University of York, York, UK.; Centre for Health Economics, University of York, York, UK; HCD Economics, Las Palmas de Gran Canaria, Spain.; Centre for Health Economics, University of York, York, UK; Janssen Pharmaceutica NV, Beerse, Belgium.; Centre for Health Economics, University of York, York, UK.; Department of Health Sciences, University of York, York, UK; Hull York Medical School, York, UK; Bradford District Care NHS Foundation Trust, Bradford, UK.; Centre for Health Economics, University of York, York, UK.

**Keywords:** England, physical health checks, primary health care, mental illness, uptake, cohort studies

## Abstract

**Background:**

People with serious mental illness are more likely to experience physical illnesses. The onset of many of these illnesses can be prevented if detected early. Physical health screening for people with serious mental illness is incentivised in primary care in England through the Quality and Outcomes Framework (QOF). GPs are paid to conduct annual physical health checks on patients with serious mental illness, including checks of body mass index (BMI), cholesterol, and alcohol consumption.

**Aim:**

To assess the impact of removing and reintroducing QOF financial incentives on uptake of three physical health checks (BMI, cholesterol, and alcohol consumption) for patients with serious mental illness.

**Design and setting:**

Cohort study using UK primary care data from the Clinical Practice Research Datalink between April 2011 and March 2020.

**Method:**

A difference-in-difference analysis was employed to compare differences in the uptake of physical health checks before and after the intervention, accounting for relevant observed and unobserved confounders.

**Results:**

An immediate change was found in uptake after physical health checks were removed from, and after they were added back to, the QOF list. For BMI, cholesterol, and alcohol checks, the overall impact of removal was a reduction in uptake of 14.3, 6.8, and 11.9 percentage points, respectively. The reintroduction of BMI screening in the QOF increased the uptake by 10.2 percentage points.

**Conclusion:**

This analysis supports the hypothesis that QOF incentives lead to better uptake of physical health checks.

## Introduction

Pay-for-performance is a healthcare reimbursement model that provides financial incentives to healthcare providers to deliver high-quality care and achieve specific performance targets. Pay-for-performance schemes have been implemented in many countries around the world including the US, the UK, Australia, Canada, and Germany, and across a range of healthcare settings,^1^ including to incentivise preventive activities in primary care.^2^ However, their effectiveness is unclear.^3^

In the UK, the Quality and Outcomes Framework (QOF) is a pay-for-performance scheme that incentivises GPs to meet specific quality targets across different aspects of healthcare delivery, such as management and treatment of specific chronic conditions, preventive care, and patient safety. In 2006, GPs were incentivised to conduct annual physical health checks on patients with serious mental illness. These patients are at increased risk of physical ill health,^4^^,^^5^ and their life expectancy is around 20 years lower than for the general population.^6^^–^8 Most premature deaths in this population are attributable to preventable causes.^9^ The rationale for incentivising physical health monitoring is that proactive physical reviews lead to earlier detection of physical health problems and trigger appropriate interventions that can prevent deterioration of health issues, and improve health outcomes. This can result in improvements in life expectancy and quality of life, and reductions in hospitalisations and costs.^10^ Medical guidelines published in the US, Australia, Brazil, Canada, and Europe have recommended physical health checks for people with serious mental illness, and some of them explicitly involve GPs in this responsibility.^11^^,^^12^

In the UK, >535 000 people with serious mental illness are registered with a GP,^13^ who oversees care, prescribes medication, and provides both mental and physical health care. In the English NHS, primary care has the lead responsibility for carrying out annual physical health checks and follow-up care for patients with serious mental illness who are not in contact with or only recently established contact with secondary mental health services, and/or whose condition has stabilised.^14^^,^^15^ Increasing the proportion of people with a comprehensive physical health review conducted in primary care is a high policy priority.^16^^–^18 The annual physical health checks incentivised by the QOF include checks on alcohol consumption, blood pressure (BP), cholesterol, body mass index (BMI), and blood glucose. However, not all of these checks were incentivised continuously (see Table 1). In the financial year 2014–2015 (running from 1 April 2014 to 31 March 2015), BMI, cholesterol, and blood glucose checks were removed from the QOF. In the financial year 2019–2020, BMI screening was re-introduced but alcohol consumption screening was removed. In financial year 2021–2022, all indicators were included in the QOF.

**Table 1.** Development of physical health checks over time

**Financial years^a^**	**2006–2007 to 2010–2011**	**2011–2012**	**2012–2013**	**2013–2014**	**2014–2015 to 2018–2019**	**2019–2020 to 2020–2021**	**2021–2022**
Annual review	Y	—	—	—	—	—	—
Alcohol consumption	—	Y	Y	Y	Y	—	Y
BMI	—	Y	Y	Y	—	Y	Y
Blood pressure	—	Y	Y	Y	Y	Y	Y
Total cholesterol	—	Y	Y	Y	—	—	Y
Blood glucose	—	Y	Y	Y	—	—	Y

a *In England, the financial year starts in April. BMI = body mass index. Y = yes.*

There is limited evidence about the effects of removing incentivised activities from pay-for-performance programmes on care delivery. A US study^19^ analysed the impact of removing financial incentives from four clinical quality indicators in Kaiser Permanente, an integrated healthcare delivery system providing comprehensive medical care to about 3.1 million people in northern California. The removal of incentives was associated with a decrease in screening rates. In the UK, research has studied the impact of removing physical health checks from the QOF on uptake using practice-level data.^20^ The authors found an immediate reduction in performance on quality measures.

However, none of these studies analyse the effect of the reintroduction of financial incentives on the uptake of physical health checks. The aim of this study was to assess the impact of removing and then reintroducing financial incentives for three physical health checks (BMI, cholesterol, and alcohol consumption) on their uptake for patients with serious mental illness. To the authors’ knowledge, this is the first study that uses patient-level data to analyse the impact of financial incentives on physical health check uptake, enabling more robust inferences on their association.

**Table:** How this fits in

Previous research has shown that removing incentives for performing physical health checks on patients with serious mental illness is associated with an immediate reduction in their uptake. This current study finds that the decrease in the uptake of three health checks (for body mass index, cholesterol, and alcohol consumption) is sustained and can only be reversed if incentives are reinstated. Despite physical health checks having been in place for a long time, it appears that they have not been fully integrated into routine practice. An implication for practice is the need to actively monitor how reductions in uptake, stemming from incentive changes, affect patients who would benefit the most from receiving these checks and follow-up interventions.

## Method

### Data

We used de-identified patient electronic health records from the Clinical Practice Research Datalink (CPRD) GOLD database, which collects fully coded routine care data from a network of GP practices using the same software system (Vision). Patients in CPRD GOLD are broadly representative of the English general population in terms of age, sex, ethnicity, and BMI.^21^ However, areas in the East Midlands, Yorkshire and the Humber, and the North East of England are under-represented in the data.

We considered all patients aged ≥18 years who were registered with a practice in CPRD GOLD at any time between April 2011 and March 2020, and had a diagnosis of schizophrenia, bipolar disorder, and other psychoses and other affective disorders documented in primary care. Although the QOF serious mental illness register is defined based on the first three diagnosis types,^22^ we included other affective disorders (constituting <4% of all serious mental illness diagnosis in our cohort) in the definition of the population with serious mental illness to broaden the scope of the QOF policy evaluation on the wider population with serious mental illness. We followed the NHS England technical guidance 2019/2020^17^ to identify GP-led physical health checks.

### Outcome

The primary outcome was a binary variable indicating whether a patient received a physical health check in a given year.

### Exposure

The exposure variable (hereafter referred to as intervention) is an indicator for whether the physical health check was subject to the policy change, that is, the removal from or reintroduction to QOF.

### Covariates

We used several covariates at a patient level: age, sex (male/female), ethnicity, years since serious mental illness diagnosis, type of serious mental illness disorder (schizophrenia, bipolar disorder, and/or other psychoses and other affective disorders), deprivation, and comorbidities.

As a measure of deprivation, we used quintiles of the deprivation level associated with a patient’s area of residence as captured by the 2015 English Index of Multiple Deprivation.^23^

Morbidities, identified using Read codes (standard clinical coding used in UK general practice),^24^ were included as binary variables indicating whether a condition had been recorded by the time patients entered each cohort. We included the following 12 conditions: asthma, atrial fibrillation, cancer, coronary heart disease, chronic kidney disease, chronic obstructive pulmonary disease, diabetes, heart failure, hypertension, chronic liver disease, rheumatoid arthritis, and stroke or transient ischaemic attack.

### Statistical analysis

To assess the impact of the removal and reintroduction of financial incentives for physical health checks on their uptake, we calculated the annual uptake rate of each physical health check in the eligible patient population registered with CPRD practices in each financial year between 2011–2012 and 2019–2020. Patients who had the same physical health check more than once in a financial year were only counted once.

We conducted a case-control study where we compared physical health checks that were incentivised for only some of the study period (cases) with physical health checks that remained incentivised throughout (controls). We employed a difference-in-differences analysis to compare differences in the uptake before and after the intervention between cases and controls. The main variables of interest were the interaction terms (formed by multiplying the exposure with time indicators), which capture the uptake changes over time of the cases relative to the controls.

The rationale for the difference-in-differences approach was that both cases and controls would be subject to the same external influences and thus exhibit similar pre-intervention time trends. Any differences in physical health check uptake observed in cases over and above the time trend of controls would therefore be attributable to the intervention (policy change).

The following QOF policy changes were analysed: the removal of BMI and cholesterol checks from the QOF in 2014–2015; the reintroduction of BMI checks in the QOF in the financial year 2019–2020; and the removal of alcohol checks from the QOF in 2019–2020. The analysis of the financial year 2014–2015 policy changes was performed in a cohort of 5635 patients (cohort-2012) who remained continuously enrolled in the same practice 2 years before and 2 years after the removal of BMI and cholesterol checks from the QOF (that is, between 1 April 2012 and 31 March 2016). BP monitoring was incentivised throughout the study period and served as control. Each patient contributed with two records in any given year: one for the case physical health check and one for the control physical health check. The total number of observations in cohort-2012 was 45 080 (5635 patients x 4 years x 2 records). The analysis of the financial year 2019–2020 interventions was performed on a cohort of 3065 patients (cohort-2018) who remained continuously enrolled in the same practice 1 year before and 1 year after the reintroduction of BMI (that is, between 1 April 2018 and 31 March 2020). The total number of observations in cohort-2018 was 12 260 (3065 patients x 2 years x 2 records). We did not include blood glucose in the analysis since there was no control with a similar pre-intervention time trend.

We estimated linear probability regression models controlling for all the above-mentioned patient-level covariates and year fixed effects. All analyses were performed in Stata (version 17).

## Results

The two cohorts were similar in terms of patient characteristics (see descriptive statistics in Table 2).

**Table 2.** Descriptive statistics: Cohort-2012 and Cohort-2018

**Characteristics**	**Cohort-2012 (*n* = 5635)**		**Cohort-2018 (*n* = 3065)**	

	**Mean**	**SD**	**Mean**	**SD**
**Sex, male**	0.49	0.50	0.52	0.50

**Age at 2012, years**	50.22	15.40	49.60	15.48

**Ethnicity, White^a^**	0.60	0.49	0.70	0.46

**Years between first diagnosis and 2012**	12.36	11.81	12.14	10.92

**Type of SMI diagnosis**				
Schizophrenia	0.65	0.48	0.64	0.48
Bipolar disorder	0.32	0.47	0.32	0.47
Other psychoses and other affective psychosis	0.04	0.19	0.05	0.21

**Index of Multiple Deprivation quintile**				
1 (least deprived)	0.17	0.38	0.15	0.36
2	0.16	0.37	0.15	0.36
3	0.19	0.40	0.19	0.39
4	0.22	0.41	0.22	0.41
5 (most deprived)	0.26	0.44	0.28	0.45

**Conditions recorded before 2012**				
Asthma	0.28	0.45	0.35	0.48
Atrial fibrillation	0.01	0.11	0.02	0.13
Cancer	0.03	0.17	0.04	0.18
Coronary heart disease	0.04	0.19	0.03	0.18
Chronic kidney disease	0.08	0.27	0.08	0.27
Chronic liver disease and viral hepatitis	0.01	0.10	0.01	0.11
Chronic obstructive pulmonary disease	0.03	0.17	0.05	0.21
Diabetes	0.10	0.30	0.12	0.33
Heart failure	0.01	0.10	0.01	0.11
Hypothyroidism	0.17	0.38	0.17	0.38
Rheumatoid arthritis	0.02	0.13	0.02	0.14
Stroke and TIA	0.02	0.16	0.03	0.17

a *Ethnic groups were categorised into two broad groups: White, Black and other minorities. SD = standard deviation. SMI = serious mental illness. TIA = transient ischaemic attack.*

**Figure 1.**
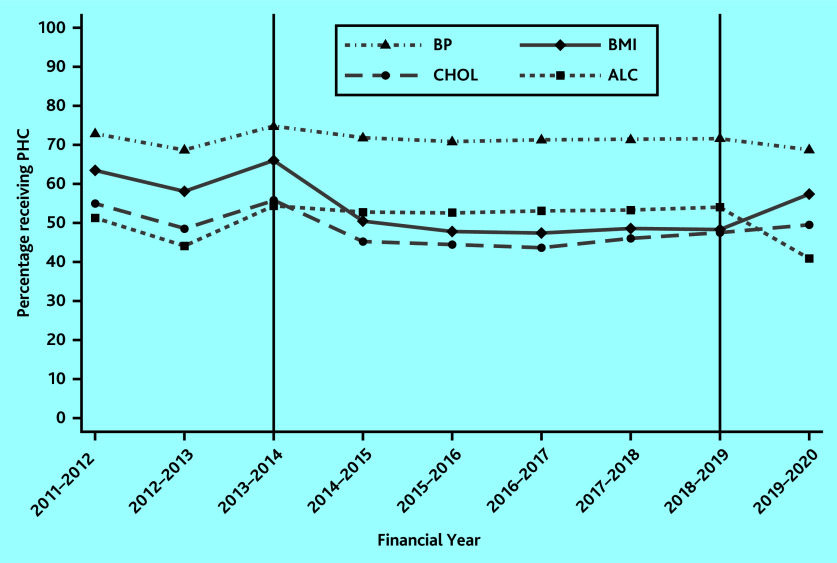
Uptake of BMI, cholesterol, BP, blood glucose, and alcohol checks over time. The first vertical line indicates the financial year prior to the removal of BMI and cholesterol checks from the QOF in 2014–2015. The second vertical line indicates the financial year prior to the reintroduction of BMI checks and the removal of alcohol checks in 2019–2020. ALC = alcohol. BMI = body mass index. BP = blood pressure. CHOL = cholesterol. PHC = physical health check.

Figure 1 shows the proportion of patients receiving BMI, cholesterol, BP, and alcohol consumption checks between financial years 2011–2012 and 2019–2020. The first vertical line indicates the financial year prior to the removal of BMI and cholesterol checks from the QOF in 2014–2015, whereas the second vertical line indicates the financial year prior to the reintroduction of BMI checks and the removal of alcohol checks in 2019–2020.

The proportion of patients receiving BMI checks dropped by 16 percentage points between 2013–2014 and 2014–2015 (from 66% in 2013–2014 to 50% in 2014–2015) and a further 2 percentage points (to 48%) in the subsequent year. This level remained constant until BMI checks were reintroduced in 2019–2020 when uptake increased to 57% (Figure 1).

The time series for cholesterol checks display a similar pattern to that of BMI checks until 2019–2020, however, with lower levels of uptake continuing to reflect that cholesterol checks were reinstated as QOF indicators only in 2021–2022 (Figure 1).

Alcohol check recording has remained stable from 2013–2014 to 2018–2019. After removal from the QOF in 2019–2020, a decrease in uptake from 54% in 2018–2019 to 41% in 2019–2020 is observed in Figure 1.

BP monitoring, which has been consistently included in the QOF throughout the period 2011–2012 to 2019–2020, exhibits the highest uptake of all physical health checks (>70%). In most years, the uptake remained unchanged, with only a small variation observed around years where QOF policy changes occurred for other physical health checks, but not for BP (Figure 1).

The level of BP, BMI, and cholesterol checks follow similar trends before the policy change (see Figure 1), a requirement for the difference-in-differences approach. Table 3 presents the estimates of the impact of removing BMI and cholesterol checks from the QOF in 2014–2015, using BP checks as a control group. The uptake of physical health checks might change over time irrespective of the introduction of the QOF policy, which is captured by the time indicators. These general temporal changes are all positive and significant, implying that the uptake of BP checks in all years is larger compared with the uptake of BP checks in 2012–2013.

**Table 3.** Impact of removal of incentives in 2014–2015 on the uptake of BMI and cholesterol physical health checks

**Cohort-2012: 2012–2016**	**BMI versus BP (95% CI)**	**CHOL versus BP (95% CI)**
Intervention	−8.4^a^ (−9.8 to −6.9)	−17.7^a^ (−18.6 to −16.7)
Baseline: 2012–2013		
2013–2014	7.3^a^ (5.9 to 8.7)	7.9^a^ (6.7 to 9.0)
2014–2015	5.1^a^ (3.8 to 6.5)	5.4^a^ (4.1 to 6.7)
2015–2016	4.0^a^ (2.2 to 5.8)	4.3^a^ (2.9 to 5.6)
DiD 2014–2015	−12.3^a^ (−14.2 to −10.4)	−6.8^a^ (−8.3 to −5.3)
DiD 2015–2016	−14.3^a^ (−16.5 to −12.0)	−6.8^a^ (−8.4 to −5.3)
Diabetes	18.2^a^ (16.0 to 20.4)	22.0^a^ (20.2 to 23.8)
Patients, *n*	5635	5635
Patient-year-PHCs, *n*	45 080	45 080

a *0.1% significance level. Confounders (other than diabetes) and practice fixed effects are omitted from the table. The full table is available from the authors on request. Intervention is a binary indicator taking value 1 if the PHC is a BMI/CHOL and 0 if it is a BP check. The figures represent percentage point change in the uptake as a result of the removal of incentives. ALC = alcohol. BMI = body mass index. BP = blood pressure. CHOL = cholesterol. DiD = difference-in-differences. PHC = physical health check.*

Differences in the uptake in the baseline year (2012–2013) between BMI/cholesterol and BP checks are given by the intervention coefficients. The uptake is lower for BMI and cholesterol checks compared with BP checks by 8.4 percentage points (95% confidence interval [CI] = −9.8 to −6.9) and 17.7 percentage points (95% CI = −18.6 to −16.7), respectively (Table 3).

The impact of the policy change on the uptake of BMI/cholesterol checks after accounting for time trends and confounders amounts to −12.3 percentage points (95% CI = −14.2 to −10.4) and −6.8 percentage points (95% CI = −8.3 to −5.3) in the first year (difference-in-differences 2014–2015), respectively. The policy change reduced the uptake of BMI checks by another 2 percentage points in 2015–2016 but did not do so for cholesterol checks (Table 3).

Table 4 presents the results of the 2019–2020 policy changes. Reintroducing BMI checks in the QOF was associated with a 10.2 percentage point increase in their uptake between 2018–2019 and 2019–2020. Removing alcohol physical health checks from the QOF list was associated with a reduction in their uptake by 11.9 percentage points.

**Table 4.** Impact of reintroducing and removing incentives in 2019–2020 on the uptake of BMI and alcohol physical health checks

**Cohort-2018: 2018–2019**	**BMI versus BP (95% CI)**	**ALC versus BP (95% CI)**
Intervention	−21.3^a^ (−25.3 to −17.3)	−15.8^a^ (−18.8 to −12.8)
Baseline: 2018–2019		
2019–2020	−2.2^b^ (−4.3 to −0.02)	−2.2^b^ (−4.3 to −0.0)
DiD 2019–2020	10.2^a^ (6.3 to 14.1)	−11.9^a^ (−16.2 to −7.6)
Diabetes	20.8^a^ (17.8 to 23.8)	14.3^a^ (11.4 to 17.3)
Patients, *n*	3065	3065
Patient-year-PHCs, *n*	12 260	12 260

a *0.1% significance level.*

b *1% significance level. Confounders (other than diabetes) and practice fixed effects are omitted from the table. The full table is available from the authors on request. Intervention is a binary indicator taking value 1 if the PHC is a BMI/ALC and 0 if it is a BP check. The figures represent percentage point change in the uptake as a result of the reintroduction (BMI) and removal (alcohol) of incentives. ALC = alcohol. BMI = body mass index. BP = blood pressure. DiD = difference-in-differences. PHC = physical health check.*

It appears that most of the morbidities included in the analysis appear to be associated with higher uptake of physical health checks (only diabetes is reported, others are available from the authors on request). Patients with diabetes are associated with the largest increases in physical health check uptake (for example, a 22 percentage point increase in the uptake of cholesterol versus BP physical health checks in cohort-2012, Table 3).

## Discussion

### Summary

The introduction of the QOF pay-for-performance incentives aimed to increase the uptake of physical health checks in primary care. However, there is no consensus on how long incentives for quality indicators should remain in place. If achievement of these indicators reaches a ceiling, there is little room for further improvement. On the other hand, it is unclear whether incentivised behaviours become so ingrained that practices continue to perform well on the indicators if incentives are withdrawn. We explored the long-term impact of removing financial incentives on the uptake of physical health checks and we provided evidence on the impact of reintroducing quality indicators in the QOF.

Our analysis supports the hypothesis that QOF incentives affect the uptake of physical health checks. We find immediate changes in the uptake of BMI, cholesterol, and alcohol physical health checks following both their removal from and reintroduction to the QOF, that are consistent with the financial incentives set under the national pay-for-performance scheme. These changes are unlikely to be explained by other changes occurring in primary care around the same time of the QOF changes, as demonstrated by our difference-in-differences analysis. Our analysis also shows that patients with chronic physical conditions such as diabetes have high physical health check uptake. This is unsurprising as patients with these conditions are more closely monitored by GPs. For instance, physical health checks such as blood pressure, BMI, and cholesterol are also incentivised for patients with diabetes.

### Strengths and limitations

This is the first study that uses patient-level data to analyse the effect of removal and reintroduction of financial incentives on the uptake of physical health checks. This allows us to make inferences on the association between the policy change and the uptake of physical health checks at patient level. Also, by controlling for patient characteristics and using a suitable physical health check as the control group, we are able to isolate changes in uptake of physical health checks that are due to the removal of incentives, from changes that are due to other factors, such as differences in patient characteristics over time and across practices, or increases in GP workload.

We used a robust methodology that allowed us to remove possible confounders and time trends that might influence the trajectory of the physical health check uptakes, and determine the causal effect.

Furthermore, our cohort design, which focused only on patients consistently registered at the same practice, prevented any loss of QOF recording data since patients who move to a new practice are exempt from QOF serious mental illness physical health check requirements for 3 months.^22^

A potential caveat of our analysis is that CPRD GOLD experienced a high rate of attrition of practices from 2013 onwards, with many practices moving away from the Vision software system.^25^ If practices that withdrew from CPRD GOLD in 2014 were more active in conducting the physical health checks under study, the large decline in the uptake of these checks from 2013–2014 to 2014–2015 could be explained by the dropout of these practices rather than the removal of physical health checks from QOF. However, in that case we would have continued to see the downward trend in physical health check uptake in subsequent years as attrition rates increased, which we did not observe.

As mentioned before, some caution is needed in generalising our results from CPRD GOLD because areas in the East Midlands, Yorkshire and the Humber, and the North East of England are under-represented in the data.

### Comparison with existing literature

Minchin *et al*^20^ compared the uptake trends of 12 QOF indicators for which financial incentives were removed in 2014 with the uptake trajectories of six other indicators for which incentives were maintained, including serious mental illness indicators for BMI, BP, and alcohol physical health checks. Their analysis was conducted at practice level for the period 2010–2017 and employed interrupted time series rather than a case–control difference-in-differences design. Their results were comparable to ours. They found similar trajectories for the uptake of BMI (a large reduction in the uptake immediately after its removal from QOF) and very small changes in the uptake of BP and alcohol checks. Minchin *et al*^20^ did not study the effect of reinstating incentives for BMI measurement, which provides further reassurance that the observed results in our study are not caused by temporal confounding but reflect causal effects of QOF incentives.

### Implications for practice

We found that removing incentivised physical health checks from the QOF has an immediate effect (decrease) on their uptake, which is sustained and only reversed if incentives are reinstated. This indicates that, despite physical health checks being in place for a long time, they have not become integrated into routine practice. While physical health checks function as ‘process indicators’ rather than ‘outcome indicators’, a decline in their uptake could hinder the early detection of health issues and reduce the likelihood of timely interventions, thereby compromising the quality of care. Consequently, practices should actively monitor the extent to which these documented losses in the uptake translate to real losses in quality of care, and try to ensure that the losses do not affect patients who are in need of receiving physical health checks. An implication for policymakers is that they should be cautious when considering policies of removing financial incentives from physical health checks for patients with serious mental illness. The physical health check uptake is very sensitive to these policies, possibly because this marginalised and disenfranchised patient group faces significant barriers to physical health check uptake. In that regard, the inclusion of all physical health checks back into the QOF in 2021–2022 (see Table 1) following a large decline in the uptake during the first pandemic year 2020–2021,^26^ appears to be a step in the right direction.

## Data

This study is based in part on data from the Clinical Practice Research Datalink (CPRD) obtained under licence from the UK Medicines and Healthcare products Regulatory Agency. The data are provided by patients and collected by the NHS as part of their care and support. The interpretation and conclusions contained in this study are those of the authors alone. The study protocol (20_000160) was approved by the CPRD’s Independent Scientific Advisory Committee.
